# Histone deacetylase 6’s function in viral infection, innate immunity, and disease: latest advances

**DOI:** 10.3389/fimmu.2023.1216548

**Published:** 2023-08-11

**Authors:** Min Qu, Huijun Zhang, Pengyuan Cheng, Ashenafi Kiros Wubshet, Xiangping Yin, Xiangwei Wang, Yuefeng Sun

**Affiliations:** ^1^ State Key Laboratory for Animal Disease Control and Prevention, College of Veterinary Medicine, Lanzhou University, Lanzhou Veterinary Research Institute, Chinese Academy of Agricultural Sciences, Lanzhou, China; ^2^ Department of Basic and Diagnostic Sciences, College of Veterinary Science, Mekelle University, Mekelle, Tigray, Ethiopia

**Keywords:** HDAC6, viral infection, innate immunity, autophagy, diseases

## Abstract

In the family of histone-deacetylases, histone deacetylase 6 (HDAC6) stands out. The cytoplasmic class IIb histone deacetylase (HDAC) family is essential for many cellular functions. It plays a crucial and debatable regulatory role in innate antiviral immunity. This review summarises the current state of our understanding of HDAC6’s structure and function in light of the three mechanisms by which it controls DNA and RNA virus infection: cytoskeleton regulation, host innate immune response, and autophagy degradation of host or viral proteins. In addition, we summed up how HDAC6 inhibitors are used to treat a wide range of diseases, and how its upstream signaling plays a role in the antiviral mechanism. Together, the findings of this review highlight HDAC6’s importance as a new therapeutic target in antiviral immunity, innate immune response, and some diseases, all of which offer promising new avenues for the development of drugs targeting the immune response.

## Introduction

Viruses are invaders that get inside the cells of many kinds of organisms, from single-celled microbes to plants and mammals. Virus infection is a prevalent cause of human sickness or death worldwide and a major source of hospital admissions in children, adults and the elderly, significantly impacting healthcare systems and human health (1, 2). The coordination of antiviral immune responses in the innate immune system, which is the first line of defense against viral infection, is required (3, 4). The innate immune system is a universal and ancient host defense against infection that recognizes nonspecific pathogens (5, 6). Most innate immune cells have molecular receptors that are genetically conserved. The immune system uses these innate immune receptors, also known as pattern recognition receptors (PRRs), including retinoic acid-inducible gene-I (RIG-I)-like receptors (RLRs), toll-like receptors (TLRs), nucleotide oligomerization domain-like receptors (NLRs), c-type lectin receptors (CLRs), and others (7). PRRs are used to identify structurally conserved molecules known as pathogen-associated molecular patterns (PAMPs), which are shared by a wide range of microbial species. Even though PAMPs from viruses, bacteria, fungi, and parasites have a wide variety of chemical structures, the immune response brought on by innate immunity cells is quick (8). Infection with a virus or an immunostimulant mimic, such as poly (I:C), can trigger the synthesis of interferon-β (IFN-β), a vital element of innate antiviral immunity (9).

HDACs are acetylation erasers from lysine residues that play critical roles in many biological processes, including immunity, cell cycle progression, apoptosis, and their repressive effects on gene transcription (10–12). HDACs have been classified into four classes in humans. Up to this point, four distinct classes of HDACs have been identified: class I (HDAC1, HDAC2, HDAC3, and HDAC8), class II (quite far subdivided into IIa comprising of HDAC4, HDAC5, HDAC7, HDAC9 and IIb consisting of HDAC6, HDAC10), class III (Sirtuins 1-7) and class IV (HDAC11) (13, 14). Except for class III, eleven of the 18 HDACs rely on Zn^2+^ for deacetylation (15). Class II HDACs are known to often move between the nucleus and cytoplasm, whereas those of class IIb HDACs are mostly localized in the cytoplasm (16). Immune cells react to invaders’ single- or double-stranded DNA or RNA in a general manner during this viral invasion. HDACs modulate this response by enhancing or weakening the results to alter genetic signatures. The JAK/STAT pathway, which is the most crucial signaling pathway against viral infection, is activated by type-I interferon (IFN-I) (17). It is noteworthy that HDAC3 interacts with forkhead box K1 to control signaling STAT1/2 transcription, which supports macrophages’ antiviral innate immunity (18). To control the expression of the interferon-stimulated gene (ISG), the released IFN cytokines first bind to IFN-I receptors and activate the JAK/STAT signaling cascade (19). HDAC4, a class II member, has anti-influenza A virus (IAV) characteristics and is a component of the host’s innate antiviral response (20). Besides, HDAC1 is crucial for the replication of the IAV, suggesting that it could be a target for innate immunity-based antiviral protection (21). HDAC6 is essential for antiviral innate immune responses (22–24). HDAC6 was first discovered due to its similarity to the *Saccharomyces cerevisiae* histone deacetylase HDA1 (25, 26). Moreover, HDAC6 is a multisubstrate enzyme that controls several cellular processes, including those that lead to cancer, neurological illnesses and inflammatory disorders. However, it is still unclear how HDAC6 is related to the antiviral signaling network and whether it is essential for antiviral immunity in animals under physiological circumstances. In particular, data show that IFN-β gene activation necessitates HDAC activity and that HDAC6 also serves as a coactivator of interferon regulatory factor 3 (IRF3)-dependent transcription (19). The failure of IFN-β production in cells is linked to the inhibition of antiviral responses (19). As a result of these factors, HDAC6 has emerged as a topic of intense interest among researchers and has become a desirable therapeutic target.

To conclude, we highlight new studies that show HDAC6’s multi-functionality in protein structure, viral infection, innate immune response, upstream signaling, and the application of HDAC6 inhibitors in associated diseases.

## HDAC6’s structure and function

**Figure 1:**
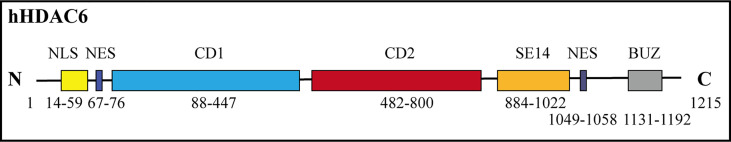
Human HDAC6’s functional domains and schematic depiction. HDAC6 has both catalytic activity and two tandem deacetylase domains (CD1 and CD2). Two nuclear export signal (NES) prevents the protein from building up in the nucleus, and the Ser-Glu-containing tetrapeptide (SE14) region guarantees persistent anchoring of the enzyme in the cytoplasm. In the nucleus, HDAC6 is translocated by the nuclear localization signal (NLS). The C terminal contains a ZnF-UBP.

**Figure 2:**
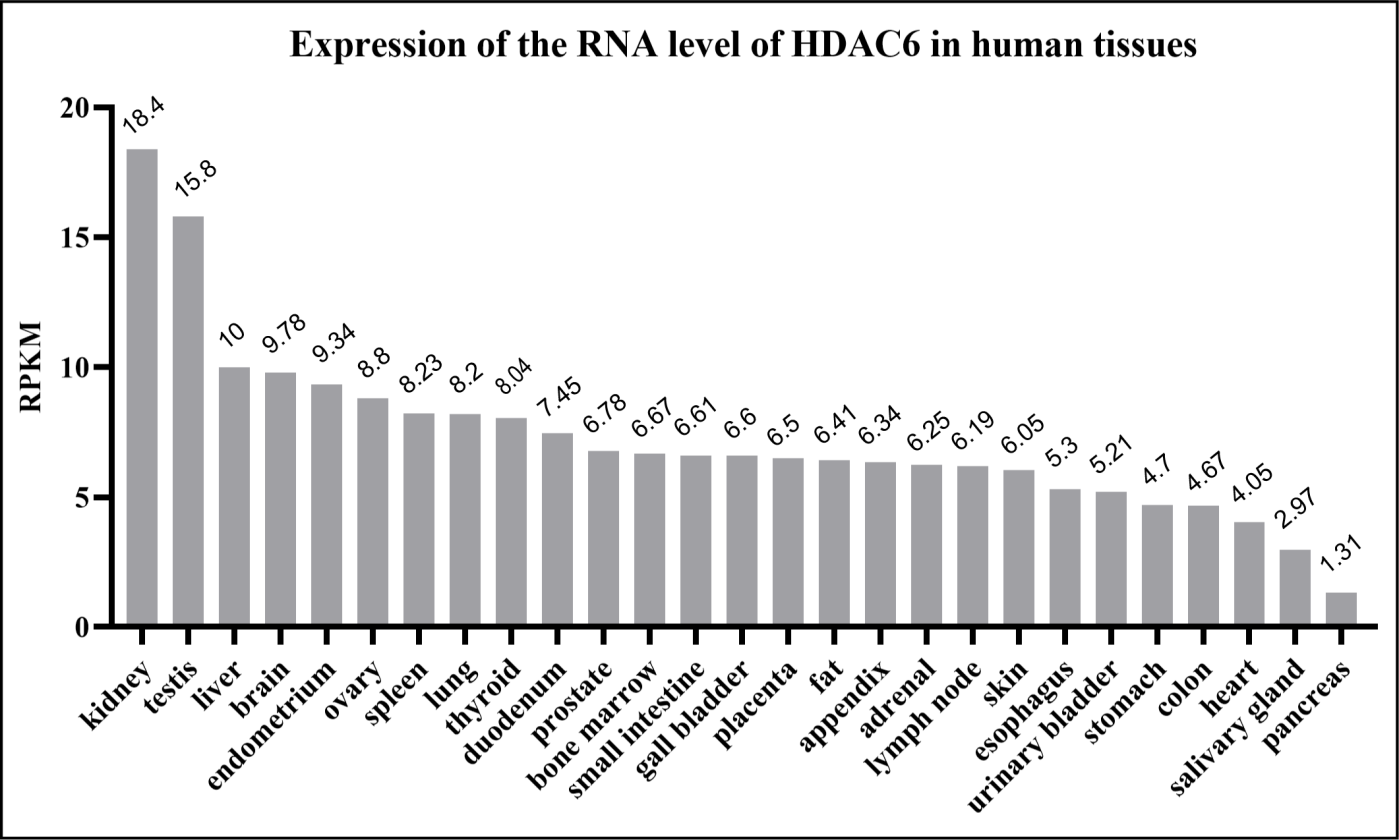
Human HDAC6 RNA expression level in normal tissues. The data from National Center for Biotechnology Information. HDAC6 is abundant in the kidney, testis, liver and brain. RPKM means reads per kilobase of exon model per million mapped reads.

The HDAC6 isoenzyme has two deacetylase domains. This enzyme is a cytoplasmic class II histone deacetylase involved in a variety of cellular functions such as immunological synapse formation, misfolded protein degradation, migration, and cell-cell contact (27, 28). With 1,215 amino acids, HDAC6 protein is the largest HDAC protein found in people. It is unique and different because it is made up of five domains. The N-terminal (1-87 aa) is made up of a nuclear-localized signal (NLS, 14-59 aa) and a nuclear export signal (NES, 67-76 aa), which together control HDAC6’s shift from the nucleus to the cytoplasm. Also, research shows that the N-terminal of HDAC6 is needed for binding and effective acetylation of tubulin (29); it has two highly conserved catalytic domains, the first of which is called catalytic domain 1 (CD1, 88-447 aa) and has been shown to have deacetylase activity (30) and ubiquitin E3 ligase activity (31); the second CD2 (482-800 aa) exhibits broader substrate specificity as a deacetylated domain with catalytic activity (32); a cytoplasmic retention signal, Ser-Glu-containing tetrapeptide (SE14, 884-1022 aa), SE14 has the ability to control how HDAC6 interacts with other proteins (33). Another NES (1049-1058 aa) and a zinc-finger ubiquitin-specific protease (ZnF-UBP domain, also known as BUZ, 1131-1192 aa) recruit ubiquitin protein to stimulate aggresome formation (**Figure 1**) (34–36). The HDAC6 gene has 21923 base pairs and is on chromosome X p11.22-23. It is found in large amounts in the testis, spermatogenic cells, germ cell tissues, liver, heart, muscle, spleen, and kidney, among other normal human tissues and organs. (**Figure 2**) (Data from https://www.ncbi.nlm.nih.gov/gene/10013) (25, 37, 38).

Also, HDAC family members, especially the cytoplasmic protein HDAC6, are very important in controlling the acetylation of non-histone (34). A proteomic analysis found at least 3,600 acetylation sites in 1,750 nuclear and non-nuclear proteins. This shows that acetylation and deacetylation play an important role in processes in both the nucleus and the cytoplasm (39). Many substrates and proteins that work with them are found in the cytoplasm, which makes sense since that is where HDAC6 was found to be concentrated. HDAC6 has been shown to interact with a number of proteins that are not histones. These include α-tubulin (40, 41), heat shock protein 90 (42), cortactin (43), peroxiredoxins (44), β-catenin (45), Ku-70 (46), Tat (47), survivin (48), extracellular signal-regulated kinase-1 (ERK-1) (49, 50), heat shock factor-1 (51), myosin heavy chain 9 (52), heat shock cognate protein 70 (52), dnaJ homolog subfamily A member 1 (52), Miro-1 (53), tripartite motif-containing protein 21 (TRIM21) (22). **Table 1** is a summary of the many HDAC6 substrates, what they do, and what viral infections they are linked to. A proteomics study found that 107 proteins in the livers of HDAC6 knockout mice could be HDAC6 deacetylating substrates (52).

**Table 1:** An overview of the effects of HDAC6 on viral infection and its substrates.

Virus	Characterization	Substrates/Targets	Deacetylation site	Effects of HDAC6	References
AdV	Nonenveloped, dsDNA	TRIM21	Lys385, Lys387	Inhibits AdV replication through deacetylating of TRIM21	(22)
COVID-19	Enveloped, (+) ssRNA	–	–	HDAC6 inhibition reduces T cell exhaustion/ACE2 expression and reduces the expression of IFN and cytokines	(54)
IAV	Enveloped, (−) ssRNA	MTs	–	Blocks IAV cellular transport by deacetylating MTs	(55)
		–	–	Be recruited to viral fusion site *via* ZnF-UBP to facilitate IAV uncoating	(56)
		PA	Lys664	Restricts IAV RNA transcription by deacetylating PA	(57)
		–	–	Disruption of HDAC6 interacts with ubiquitin to impair IAV infection	(58)
HCV	Enveloped, (+) ssRNA	–	–	Deacetylation activity induces RIG-I signaling to inhibit HCV infection	(59)
HIV	Enveloped, (+) ssRNA	Tat	Lys28	Inhibits HIV-1 replication by deacetylating Tat and reducing its transactivation ability	(47)
		Tat	–	Promotes HIV-1 Tat-induced proinflammatory responses by activating MAPK-NF-κB/AP-1 pathways	(60, 61)
		tubulin	–	HDAC6 inhibition improves axonal transport and protects the HIV-1 envelope protein gp120 from neurotoxicity	(62)
		α-tubulin	–	Inhibits HIV-1 fusion and infection by deacetylating α-tubulin	(63)
		Pr55Gag/ Vif	–	Inhibits HIV-1 production by promoting the degradation of viral proteins Pr55Gag and Vif	(64)
HIV	Enveloped, (+) ssRNA	α-tubulin	–	TDP-43 promotes HDAC6 expression and inhibits HIV-1 entry and infection	(65)
HPIV3	Enveloped, (−) ssRNA	α-tubulin	–	Blocks HPIV3 fusion by deacetylating α-tubulin	(66)
oHSV	Enveloped, dsDNA	–	–	Inhibits oHSV infection by promoting viral autophagy	(67)
PCV2	Nonenveloped, ssDNA	cGAS	–	Be recruited to promote cGAS degradation and inhibit IFN-I secretion to promote PCV2 infection	(68)
SeV	Enveloped, (−) ssRNA	β-catenin	Lys49	Inhibits SeV infection by upregulating IFN-β expression	(19, 24, 69)
VSV	Enveloped, (−) ssRNA	–	–	Inhibits VSV infection by upregulating IFN-β expression	(19, 24)

(–) refers to negative strand RNA virus, – in the substrates/targets and deacetylation site refers to none.

HDAC6’s tubulin deacetylase activity was initially reported by Hubbert et al. in both *in vivo* and *in vitro* settings (40). In the end, it was found that the activity of HDAC6 recombinant mutants is controlled by mutations in certain catalytic domains. Their analysis showed that *in vitro* deacetylase activity was only encountered in the CD2 domain (35). In contrast to other HDACs, HDAC6 has a Cys/His-rich C-terminal region that interacts with polyubiquitin in a novel way while preserving the deacetylase activity of the protein (37). It has been reported that HDAC6 inhibitors regulate viral infection, tumorigenesis and disease development. Specific HDAC6 inhibitors are known so far including Tubacin, Tubastatin A, ACY-1215, ACY-24, Nexturastat A and others (34). HDAC6 inhibitors are gradually being used as a novel strategy for antiviral and antitumor drug development.

## Upstream antiviral signaling promotes HDAC6

**Figure 3:**
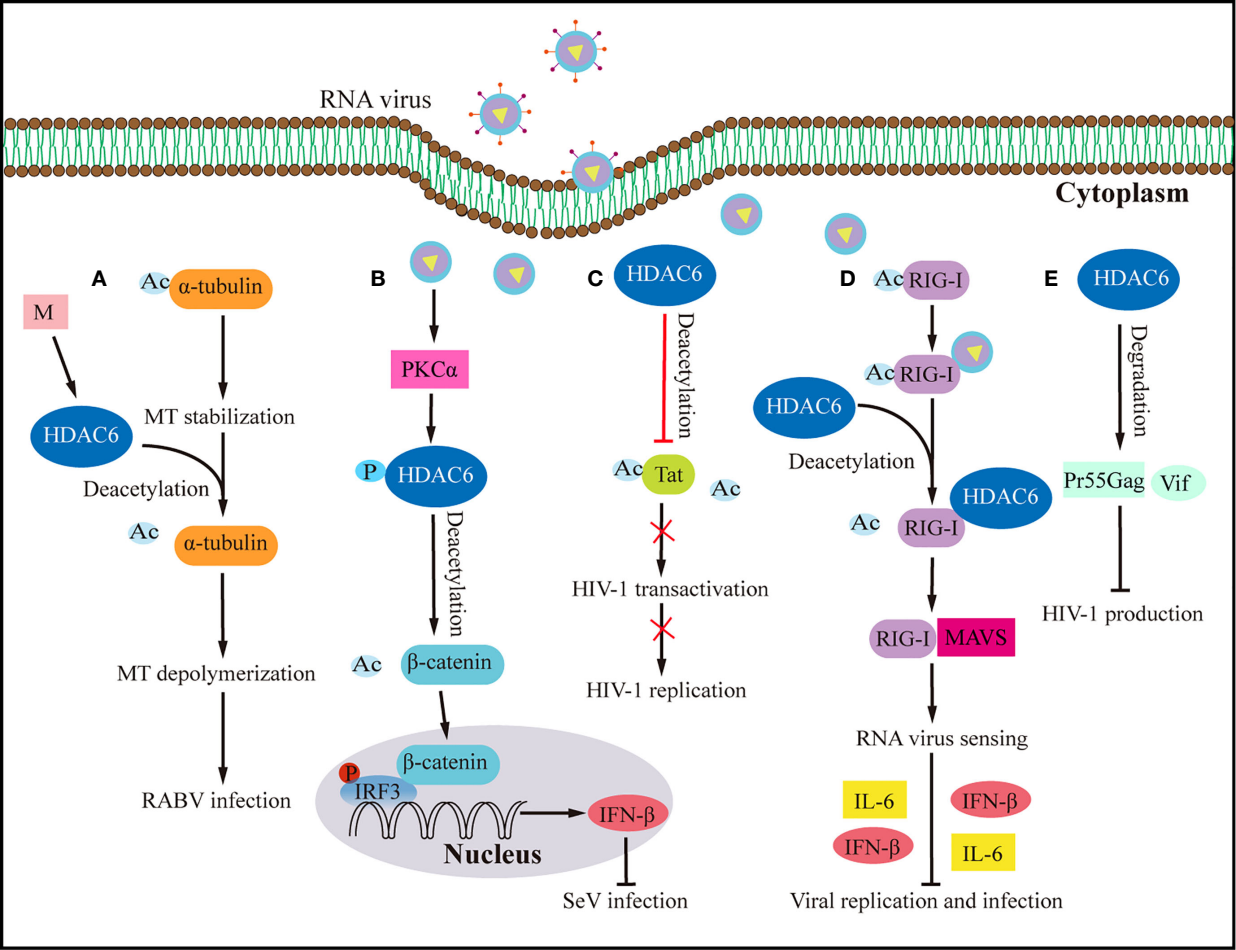
Effects of HDAC6 on RNA virus infection and host antiviral immunity. **(A)** By upregulating HDAC6, the M protein of the rabies virus induces MTs depolymerization to facilitate viral RNA synthesis. **(B)** HDAC6 deacetylation of β-catenin promotes nuclear translocation and IRF3-mediated IFN-β transcription, thereby inhibiting the infection by SeV. **(C)** HDAC6 prevents HIV-1 replication by deacetylating Tat and decreasing its ability to transactivate. **(D)** By interacting with RIG-I and promoting RIG-I deacetylation and sensing of RNA viruses, HDAC6 accelerates the transmission of the RLR signaling pathway when it is overexpressed, which increases the production of IFN-β and inflammatory proteins to prevent viral infection. **(E)** HDAC6 prevents the production of HIV-1 by promoting the degradation of viral proteins Pr55Gag and Vif. Ac, acetyl; M, matrix protein of rabies virus; P, phosphorylation; RABV, rabies virus.

The host antiviral response depends on understanding HDAC6’s upstream signaling molecules. Transactive response DNA-binding protein 43 kDa (TDP-43), a nuclear RNA binding protein, is important in RNA processing (70, 71). TDP-43 particularly binds HDAC6 RNA, according to current research (65). TDP-43 stabilises the antiviral enzyme HDAC6 and increases its mRNA and protein levels, the study found (65). The upregulation of MT acetylation and the downregulation of the antiviral HDAC6 enzyme in response to low TDP-43 levels make target cells more vulnerable to human immunodeficiency virus (HIV) infection (65). High TDP-43 levels, on the other hand, change the cellular level of the HDAC6 antiviral factor, preventing HIV-1 envelope complex (Env) fusion and infection regardless of viral Env tropism and strain (65). The acetylation of α-tubulin lysine-40 amino acids is considered a sign of microtubules (MTs) stability (72), and as early as 2022, researchers found that HDAC6 can deacetylate α-tubulin (73). Rabies is caused by the Rabies virus, which has a high fatality rate and offers a significant health risk to humans. It was shown that expressing the rabies virus M protein alone significantly increased HDAC6 expression, leading to a significant decrease in its substrate, acetylated-tubulin, and ultimately MTs depolymerization (74) (**Figure 3A**). MTs depolymerization increases viral RNA production substantially (74). Inhibiting HDCA6 deacetylase activity drastically reduced rabies virus RNA synthesis, indicating that HDAC6 plays an essential role in viral infection. The protein kinase C (PKC) family is a type of serine/threonine kinase that is activated by phospholipids and calcium (75). PKCα is a member of the PKC family that is implicated in cancers, viral infection, and other signaling pathways (75, 76). Although IRF3 activation controls interferon synthesis, a recent study has demonstrated that IRF3 also works in conjunction with the protein β-catenin (69). A previous study has shown that HDAC6 activates β-catenin by deacetylation (69). However, following viral infection, how β-catenin was activated to affect downstream signaling pathways is still elusive. Zhu et al. (24) demonstrated a signaling pathway involving sendai virus (SeV) infection activated PKCα, which increases the phosphorylation of HDAC6, which then deacetylates β-catenin and promotes its translocation to the nucleus, promotes the phosphorylation of IRF3 and the production of IFN-β, which eventually lead to the inhibition of SeV replication (**Figure 3B**).

## HDAC6 mediates viral infection as well as host innate immunity

HDAC6 regulates both DNA and RNA viral replication, with recent research concentrating on influenza viruses among RNA viruses. HDAC6 mainly confronts virus invasion by controlling host cytoskeleton MTs to dynamically control virus transport, fusion, or uncoating. HDAC6 can also control viral infection by modifying the human immune system and autophagy. HDAC6’s upstream signaling pathway and its downstream target in anti-viral signaling, regrettably, remain largely unknown. Recent research on HDAC6 activity emphasizes its importance in the innate immune response to viral infection. Studies on HDAC6’s function in the infection of viruses have mostly focused on three aspects: (a) controlling viral transport, fusion, and viral component release by modulating plasma membrane dynamics and the cytoskeleton (55, 56, 62, 63, 74, 77); (b) influencing host cells’ antiviral immune response (19, 23, 69); (c) tuning the breakdown of viral or host proteins in host cells through autophagy (64, 68, 78).

### HDAC6 regulates viral infection by changing the cytoskeleton and dynamics of plasma membranes

Through its effect on the cytoskeleton, HDAC6 controls viral transcription, replication, entry, and the movement of viral parts. This can be good or bad for a viral infection (55, 74). MTs have been known for a long time to be important in how the cytoskeleton moves during viral infection (79). It has been shown that viruses not only change MT and MT-related proteins to help them infect cells, but they also cause specific post-translational modifications (PTM) to help them spread through cells and cause side effects of how they spread (80). Acetylation of lysine is a reversible PTM that affects many cellular processes, such as chromatin remodeling, signaling, RNA splicing, gene expression, the cell cycle, protein stability, and protein transport (81). It happens at the lysine-amino ends, which are watched over by both histone acetyltransferases (HATs) (82) and HDACs (83). Some HATs and HDACs have been found to be very important in controlling IFN-I production and response (19, 24, 84). In 2002 and 2003, researchers found that HDAC6-mediated deacetylation was linked to the stability of MTs dynamics *in vivo* and that HDAC6 deacetylated both tubulin and MTs (41, 73). The cytoskeleton controls how the host cell membrane moves, which is how animal viruses get in (85). The host cytoskeleton is made up of microfilaments, MTs, and intermediate filaments (86, 87). These filaments guide a number of processes, such as reshaping plasma membranes, capturing and moving cargo, and arranging organelles in space, which are important for cell shape, polarity, movement, or division. Many viruses use the MT-based transport system of the host cell to move around inside the cell (88, 89).

**Figure 4:**
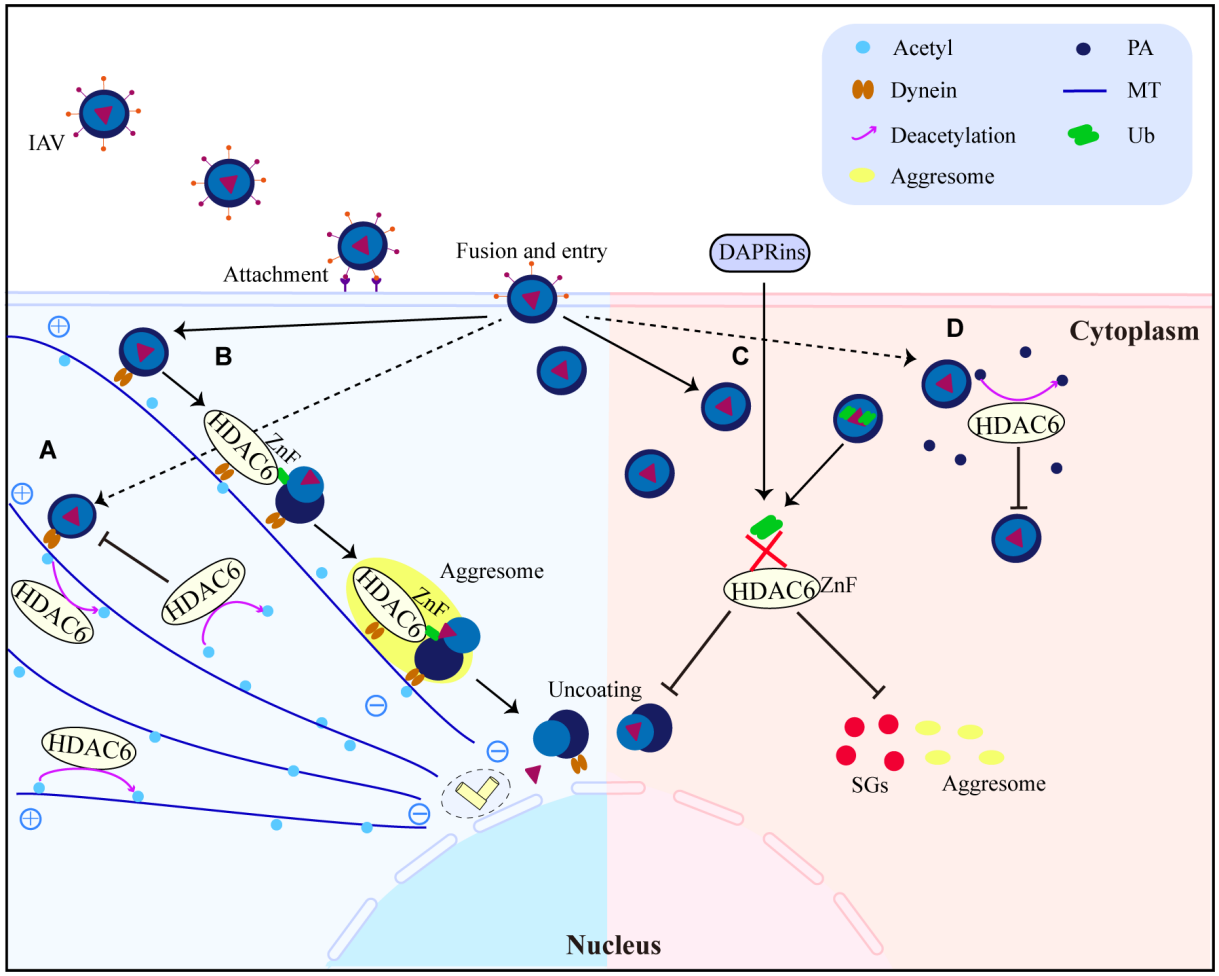
HDAC6 promotes or inhibits IAV replication and infection interestingly. **(A)** HDAC6 inhibits IAV release by downregulating IAV viral component transport by deacetylating MTs. **(B)** HDAC6 interacts with dynein, IAV can promote virus uncoating by utilizing the HDAC6-Znf-dependent aggresome formation mechanism. **(C)** DARPins block the binding of HDAC6-ZnF to Ub, inhibit the production of downstream SGs and impair IAV infection. **(D)** Through the destabilization of PA, HDAC6 functions as a negative regulator of IAV infection. HDAC6 binds to and deacetylates PA, promoting its proteasomal degradation. PA, polymerase acidic protein; MT, microtubule; Ub, ubiquitin.

HDAC6 is a major regulator of the invasive pathway of viruses. It affects how MTs move and how T regulatory cells work (40, 90), and it is a key part of how IAV gets infected (57). Seasonal outbreaks of IAV continue to kill a lot of people and make a lot of people sick all over the world. IAVs from birds can spread to humans and change enough to cause a pandemic (91). There is still a chance of a future IAV pandemic because new bird IAV strains (like H10N8, H6N1, H7N9, H9N2, H1N1, and H5N6) keep showing up in humans. IAV has already caused a pandemic this century (92–97). IAV attacks the airway epithelium of a person’s respiratory system to start the infection, which then leads to the flu, an acute fever-related respiratory illness. It has been shown that acetylated MTs help IAV nonmembrane components move (55). However, the deacetylation of MTs by HDAC6 prevents the trafficking of IAV components (55). HDAC6 is an anti-IAV host factor that works by using acetylated MTs as its substrate to inhibit the movement of viral components toward the plasma membrane of the host cell (55) (**Figure 4A**). However, when in IAV infection, the virus can take advantage of a variety of host characteristics to aid in its reproduction. For example, to aid uncoating and infection, IAV can use the aggresome-processing machinery driven by HDAC6 (56). IAV hijacks host HDAC6 to employ the host’s aggresome pathway to uncoat itself during viral entry (56). The ubiquitin-binding domain, but not the deacetylase activity, was needed for HDAC6 to be brought to viral fusion sites and for viruses to uncoat and infect cells (56). A further study showed that HDAC6 interacts with MT motor protein cytoplasmic dynein and its activator dynactin to bind unanchored ubiquitin chains that mimic unfolded proteins in the capsid, assisting in its transit to the aggresome (56) (**Figure 4B**). But new research has discovered that interfering with the interaction of HDAC6 and ubiquitin impairs IAV and Zika virus infection (58). It suggests that designed ankyrin repeat proteins (DARPins), which can block the ZnF pocket where ubiquitin engages, also inhibit aggresomes and stress granules (SGs) formation (58) (**Figure 4C**). The effects of acetylation and deacetylation on viral proteins and human immune pathway proteins impact viral replication. Other RNA viruses, like the HIV-1, use the MTs for their own purposes, just like IAV does. For instance, MTs were found to be crucial for HDAC6 to interact with Tat and for HDAC6 to deacetylate Tat and stop HIV-1 transactivation (47) (**Figure 3C**). HDAC6 reduction promotes axonal transport and avoids HIV-1 envelope protein gp120 neurotoxicity (62). HDAC6-mediated deacetylation of α-tubulin, for example, inhibits HIV-1 fusion and infection (63). Recent research has demonstrated that the suppression of HDAC6 greatly increases the amount of acetylation of α-tubulin, which enhances the fusion and reproduction of human parainfluenza virus type 3 (HPIV3) (66). TANK-binding kinase 1 (TBK1), an IKK-related serine/threonine kinase, plays a big role in antiviral immunity (98–101). Recent research shows that HDAC6 controls the phosphorylation of TBK1 and Akt in macrophages by poly (I:C) (23). Reports show that when double-stranded RNA (dsRNA) binds to TLR3, it turns on TBK1 and starts signaling transduction that leads to the production of IFN-I (98–101). The cell signaling molecule Akt also interacts with MT (102, 103). It has been observed that Akt regulates glycogen synthase kinase-3β (GSK-3β) activity and interleukin-10 (IL-10) expression (104, 105). GSK-3β has a pivotal role in controlling TBK1 activity (106). In the presence of a virus, GSK-3β interacts to TBK1, leading to the phosphorylation of TBK1 (106). Numerous studies indicate that MTs and GSK-3β have intimate interactions (107–109). Poly (I:C) induced IFN-β expression in macrophages was elevated after HDAC6 deletion, which influenced IFN-I and IL-10 production *via* enhancing TBK1 activity and removing GSK-3β inhibitory regulation (23). The results of the experiments with HDAC6 knockout mice highlight the effect of HDAC6 deletion on the immune system, confirming the importance of HDAC6 in viral infection. HDAC6 knockout mice exhibited abnormalities in bone homeostasis and immunological function (38). The number of IFN-I and IL-6 produced by these mice is also lower, making them more vulnerable to lethal RNA virus infection (110).

Generally, these results highlight the complexity of the relationship between hosts and invaders by showing that HDAC6 can have both positive and negative effects on viral replication and indicating that effective HDAC6-targeting techniques may fundamentally vary between various viral species.

### HDAC6 influences the host’s antiviral immunological response

HDAC6 acts as an anti-IAV host factor by deacetylating polymerase acidic protein (PA), which reduces IAV RNA polymerase activity (**Figure 4D**) (57). A recent study found that HDAC6 mutant mice have a higher proclivity to produce IAV infection (111), emphasising the importance of HDAC6 in anti-IAV infection. In embryonic stem cells and animals, HDAC6 overexpression enhances viral resistance (112). According to the latest research, TRIM21 is a substrate of HDAC6, and acetylation regulates its function (22). HDAC6 interacts with TRIM21 *via* the PRYSPRY motif and deacetylates it at lysines 385 and 387, facilitating homodimerization (22). Enhanced TRIM21 acetylation caused by HDAC6 inhibition, together with the suppression of TRIM21 dimerization and ubiquitination caused by hyperacetylation, prevents TRIM21 from adhering to the antibody-bound adenovirus (AdV) type 5 complex and being destroyed by the ubiquitin-proteasome pathway (22). HDAC6 depletion or inhibition promotes virus accumulation in cells, indicating a reduced ability for virus intracellular neutralization mediated by antibodies (22), which confirmed that HDAC6 is a new activator of TRIM21-mediated anti-viral innate immune response. Furthermore, HDAC6 deletion suppressed poly (I:C)-induced Akt activation, as evidenced by decreased phosphorylation of Akt at Ser473 near the carboxy terminus, implies that HDAC6 might control the degree to which macrophages activate Akt during viral infection (23). The findings imply that HDAC6 may modulate macrophage innate immunological responses to viruses.

RLRs, which include RIG-I and MDA5, are critical in innate immune responses to viral infection (113–117). RLRs in the cytosol detected invading RNA viruses (118). MDA5 and RIG-I detect different dsRNAs, MDA5 recognizes poly(I:C), and promoting production of IFNs in response to paramyxoviruses, influenza virus, and RIG-I recognizes *in vitro* transcribed dsRNAs and picornavirus (119). RIG-I containing CARDs and DExD/H box helicase domain and inducing production of IFNs in case of infection with dsRNA (113). RIG-I is required for viral RNA recognition and activation of downstream signaling pathways (110), such as the SeV (120), IAV and vesicular stomatitis virus (VSV) (121). RIG-I has piqued the interest of researchers as a viral sensor, able to distinguish 5’-triphosphate-containing dsRNA from a range of viruses or short dsRNA molecules (113, 114, 122). Additionally, overexpression of HDAC6 but not the catalytically inactive mutant boosted RLR-mediated RNA virus replication decrease (110). Binding of K63-linked polyubiquitin chains to RIG-I and start assembly of mitochondrial anti-viral-signaling protein (MAVS; also known as VISA, CARDIF, and IPS-1) activates TBK1 and IKK through a K63 polyubiquitin-dependent mechanism (123). Subsequently, transcription factors IRF3 and nuclear factor kappa B (NF-κB) are activated, and type I interferons and pro-inflammatory cytokines are turned on (123). HDAC6 regulates deacetylation of the RIG-I C-terminal domain, which limits the protein’s capacity to recognize viral RNA (110). HDAC6 acts as a deacetylase that enhances RIG-I activation and innate antiviral defense in order to identify and reduce hepatitis C virus (HCV) and other RNA virus infections (59). HDAC6 temporarily bound to RIG-I in the presence of viral RNAs, deacetylating lysine 909 (K909) to improve RIG-I’s ability to detect viral RNAs (**Figure 3D**) (110). Acetyl-mimicking mutants of RIG-I do not induce virus-induced assembly of active homo-oligomers, whereas deacetylation of RIG-I promotes the oligomerization of RIG-I and binding to the ligand MAVS (**Figure 3D**) (59). Reduced HDAC6 expression led to compromised defense against RNA viruses but not DNA viruses (110). Reducing or eliminating endogenous HDAC6 in immune cells increased viral multiplication and lowered IFN-I and pro-inflammatory cytokine production in response to RNA viruses (110). IFN-I responses are essential for the host innate immunity to protect against infections (124, 125). When cytoplasmic RIG-I-like receptors detect viral RNA, they send signals to the transcription factors IRF3 and NF-κB, which in turn induce IFN-I transcription (126). MAVS binds to RIG-I upon virus recognition and stimulates the production of ISGs by activating NF-κB and other interferon regulatory factors (127, 128). In order to offer antiviral defense and regulate immunity to infection, this mechanism leads to the development of MAVS, which in turn activates NF-κB and IRF3 signaling pathways and triggers the production of IFN-I and a large number of antiviral and immunology-related genes (129). In response to viral infection, RIG-I or TLR3 signaling activated IRF3, a critical transcription factor for IFN induction (130, 131). It has been demonstrated that HDACs participate in the TLR signaling pathway of the innate immune system. For instance, HDAC6 facilitates ubiquitin-dependent myeloid differentiation primary response 88 (MyD88) aggregation, which hinders TLR4 responses and prevents a viable MyD88 signaling platform from emerging (132). In addition, the intracellular bacterium Listeria monocytogenes activates HDAC6 to regulate innate immune and autophagy responses to TLR-mediated signals (133). Ablation of HDAC6 appears to decrease NF-κB activation in response to TLR stimulation and permit faulty autophagy, resulting in impaired bacterial clearance. HDAC6 is linked to the TLR adaptor protein MyD88 (133).

In conclusion, these findings imply that enhancing HDAC6 responses may protect against viral infection and effectively demonstrate that HDAC6 may improve the host IFN-I response to obstruct viral pathogenesis. HDAC6, a host factor, acts as an antiviral target by modulating viral transport, uncoating, replication, and infectivity, as well as IFN-I expression.

### HDAC6 controls viral infection by degrading host or viral proteins

In addition to controlling RNA viral infection, HDAC6 affects the host’s innate immune response following DNA virus infection. Porcine circoviruses (PCV) are non-enveloped tiny viruses with single-stranded circular DNA genomes (1.76 kb) that belong to the Circoviridae family (134, 135). PCV2 is a type of PCV that promotes cyclic GMP-AMP synthase (cGAS) phosphorylation at S278 in the early stages of infection by activating phosphatidylinositol 3-kinase (PI3K)/Akt signaling, which directly silences cGAS catalytic activity (68). Then, by the activity of the HDAC6 deacetylase, cGAS phosphorylation at the S278 site can stimulate k48-linked polyubiquitination at the K389 site, which can be exploited as a signal and facilitate the translocation of k48-ubiquitinated cGAS from the cytosol to the autolysosome (68). According to the results, PCV2 inhibits IFN-I induction to enhance DNA virus infections by targeting cGAS (68). As such, TRIM14 recruits USP14 to cleave the K48-linked ubiquitin chains of cGAS at K414 during herpes simplex virus type 1 (HSV-1) infection (136). This inhibits the p62-mediated autophagic degradation of cGAS and increases the activation of IFN-I signaling (136). ISG15 association with HDAC6 and p62 and it was activated by type I IFN signaling, which enhances the activity of selective autophagy, resulting in the effective clearance of ubiquitin and ISG15-tagged unwanted proteins and pathogens *via* p62 and HDAC6 (137). ISG15-mediated protein conjugation may therefore be the IFN system that permits the cell to utilize autophagy as an innate antiviral defense (137).

To inhibit HIV-1 production, HDAC6 promotes aggresome/autophagic degradation of the viral polyprotein Pr55Gag (64). By targeting the HIV proteins Pr55Gag and viral infectivity factor (Vif), HDAC6 functions as an anti-HIV-1 limiting factor, reducing viral proliferation and infection (64) (**Figure 3E**). SGs are dynamic structures that can be targeted for autophagic clearance, which is known as granulophagy (138). Aside from that, granulophagy may be an approach used by viruses to suppress antiviral immune responses (78). SGs mentioned above can serve as an antiviral immune complex and play a positive role in IFN-I response, while HDAC6 is also a component of SGs (78). In recent years, studies have found that coxsackie virus A16 infection leads to granulophagy, degradation of SGs, and selective autophagy inhibits IFN-I response (78). The findings demonstrate that viral replication and proliferation can be prevented by limiting granulophagy. Furthermore, HDAC6 inhibits oncolytic herpes simplex virus (oHSV) replication in glioma cells by aiding oHSV endocytic entry and subsequent fusion to lysosomes, hence steering incoming virions to autophagy/xenophagy rather than viral multiplication in the nucleus (67). HDAC6, a ubiquitin-binding deacetylase that targets protein aggregates and damaged mitochondria, is a key component in basic autophagy (139). HDAC6 activates autophagy by activating a cortactin-dependent actin-remodeling mechanism, which then assembles an F-actin network that promotes autophagosome-lysosome fusion and substrate degradation (139). HDAC6 is involved in the regulation of autophagy, whether as an antiviral factor to promote viral protein degradation or as a component of an antiviral complex to be degraded by viruses, which provides new ideas for host antiviral strategies.

## Diseases involving HDAC6

More attention has been paid to HDAC6’s many roles in pathology and physiology because it could be used to treat a wide range of diseases. Recent studies and reports have shown that selective HDAC6 inhibition is a good way to fight neurodegenerative diseases like Alzheimer, Huntington, and Parkinson (140). The cells of the innate immune system are the most important line of defense against pathogens and cancer cells. HDAC6 is closely linked to a number of diseases that are linked to cancerous tumors (141). As well as cancers like lung cancer, breast cancer, and ovarian cancer (28). In the case of hepatocellular carcinoma (HCC), pro-inflammatory cytokines increase the amount of HDAC6 in the body. This can cause more cells to grow by stopping p53 from doing its job as a transcription factor and causing it to be broken down (142). Hepatitis B virus (HBV), as a stronger inducer of HCC, chronic infection of HBV increased risk of HCC (143, 144). According to research, the protein expression levels of HDAC6 in HBV patients’ serum and liver decreased significantly after antiviral treatment (145). It has been reported that HBV internalization is sparked by the host-entry cofactor epidermal growth factor receptor (146). A deficiency of HDAC6 promotes epidermal growth factor receptor endocytic trafficking and degradation (147). In addition, the study found that HDAC6 can promote human papillomavirus (HPV) positive cervical cancer by upregulating Wnt5a (148). In the small-cell lung cancer xenograft model, HDAC6 was found to be a possible treatment target when used with JQ1 (149). More research needs to be done to find out if HDAC6 overexpression mice cause tumors or if they are more or less likely to get tumors. The innate immune response is different and ends with the production of cytokines (150). A macrophage releases ILs, cytokines that give instructions to other immune cells when it encounters a virus, bacteria, or other pathogens (151, 152). The innate immune response is a quick, nonspecific reaction that is controlled by a number of molecules. HDACs are important for controlling the size and strength of the response, which makes it possible to make just the right amount of inflammatory cytokines and ILs (153). Early-onset familial Parkinson’s disease is caused by changes in the protein Parkin, which is a ubiquitin ligase (154). Parkin increases mitophagy by accelerating mitochondrial ubiquitination, which attracts ubiquitin-binding autophagic components, HDAC6 and p62, resulting in mitochondrial clearance (154). Tubulin deacetylation is caused by the enzymatic activity of HDAC6 (40), and blocking tubulin deacetylation is effective in avoiding neurodegeneration in animal models of Alzheimer’s and Huntington’s disease (155, 156).

Through the transcriptional activation of their respective genes by a transcription factor NF-κB, which is a crucial component of the immune response, these cytokines are frequently produced by a variety of innate immune cells, HDAC6 has a role in HIV-1 Tat-induced pro-inflammatory gene expression by regulating the mitogen-activated protein kinase NF-κB/AP-1 pathways and it is a biological target for HIV-1 Tat-mediated neuroinflammation (60, 61). Interestingly, HDAC6 is also involved in the severe acute respiratory syndrome-coronavirus-2 (SARS-CoV-2) pandemic that is currently underway (coronavirus disease 2019; COVID-19) (157). HDAC6 inhibition has the potential to be used to minimize the morbidity associated with severe COVID-19 *via* modulating the innate and adaptive immune systems (54). HDAC6 inhibition, which blocks IFN-I synthesis and its downstream effects in airway epithelial cells and immune cells, may be able to mitigate the negative effects generated in severely ill COVID-19 patients by late or extended activation of the IFN-I pathway (54). These results support the use of HDAC inhibitors as part of an epigenetic therapeutic strategy in the treatment of severe COVID-19 (54).

HDAC6 may be a risk factor for tuberculosis (TB) and a novel host-directed anti-TB therapeutic target (158). HDAC6 also affects allergic skin inflammation. By directly modulating sirtuin 1 expression, HDAC6-negative MiR-9 prevented atopic dermatitis (159). HDAC6 and CXCL13 influence cellular connections and miR-9 and SIRT1 expression (159). HDAC6 regulates inflammation. HDAC6 reduces NACHT, LRR, and PYD domains-containing protein (NLRP3) inflammasome activity by interacting with ubiquitinated NLRP3 (160). HDAC6 knockout mice develop normally, but their immune systems are weakened (38). HDAC6 promotes monocyte/macrophage infiltration during inflammation and suppresses T cell IL-17 production, providing new insights into its role in the immune system (161, 162). A recent study demonstrated that administering an HDAC6 degrader to LPS-induced mice reduced the activation of the NLRP3 inflammasome, confirming for the first time that HDAC6 proteolysis targeting chimera may be a potential treatment for NLRP3-related illnesses (163).

## The application of HDAC6 inhibitors

Tubacin, as a selective inhibitor of HDAC6, plays a positive role in anti-viral and anti-tumor diseases (164–166). It is worth noting that “tubacin”, which prevents α-tubulin deacetylation in mammalian cells, was discovered for the first time using an omnifarious, chemical genetic screen of 7,392 small molecules (167). In the early years, as an HDAC6 selective inhibitor, tubacin induces apoptosis in epstein-barr virus (EBV)-burkitt lymphoma cells and kills EBV lymphoblastoid cells by producing reactive oxygen species (165). A few years ago, researchers found that tubacin reduces Japanese encephalitis virus replication by reducing viral RNA synthesis (164). The role of inhibiting viral replication was also demonstrated by HDAC inhibitors including suberoylanilide hydroxamic acid (SAHA) (168), which improves the susceptibility of uninfected CD4+ T cells to HIV by boosting the kinetics and efficiency of postentry viral processes (169). On the other hand, HDAC6 knockout mice can live and grow normally, tubulin hyperacetylation does not interfere with normal mammalian development, indicating that HDAC6 inhibitors could have less negative effects (38), as opposed to the inhibition of specific class I HDACs and other HDACs. These results encourage more research into the efficacy of HDAC6 inhibitors in a variety of diseases, including hematological malignancies (170, 171), neurodegenerative diseases (140) and cancers (28). Moreover, specific inhibition of HDAC6 normalizes B cell activation and germinal centre development in a model of systemic lupus erythematosus (172). These tests will provide the basic science foundation for moving forward with clinical trials of an inhibitor that selectively targets HDAC6. There is great potential for HDAC6 as a target molecule in clinical drug research, as more than a dozen HDAC6s are already in use for the treatment of various disorders.

## Conclusions

This review summarizes the function of HDAC6 in viral infection, host antiviral immunity and immune-related disorders. HDAC6 is a cytoplasmic class II histone deacetylase involved in various cellular functions, including immunological synapse formation, misfolded protein degradation, migration, and cell-cell contact. HDAC6 has two highly conserved catalytic domains: catalytic domain 1 (CD1) and CD2, which exhibit broader substrate specificity as a deacetylated domain with catalytic activity. Another NES (1049-1058 aa) and a ZnF-UBP domain recruit ubiquitin protein to stimulate aggresome formation. The HDAC6 gene has 21923 base pairs and is on chromosome X p11.22-23. It is found in large amounts in the testis, spermatogenic cells, germ cell tissues, liver, heart, muscle, spleen, and kidney, among other normal human tissues and organs. HDAC6 functions as a host antiviral factor to prevent the invasion and infection of invaders, while the virus will utilize some HDAC6 properties to escape the host’s defensive response and aid in the virus’s intracellular cycle completion. HDAC6 can reduce IAV release by deacetylating MTs, inhibit viral transcription by inhibiting the IAV RNA polymerase PA subunit, and assist viral uncoating *via* the aggregation formation mechanism of HDAC6’s ZnF domain. Blocking HDAC6 binding of ZnF to ubiquitin can decrease aggregation formation, reducing IAV uncoating. HDAC6 is involved in the process of a range of viral infections and can be exploited as a potential therapeutic target for many disorders. Therefore, the review’s findings show that HDAC6 is an attractive and new potential therapeutic target for the development of immune-enhancing medicines in the areas of antiviral immunity, innate immune response, and certain diseases.

## Author contributions

MQ and XW conceived and drafted the manuscript. MQ, YS, AW and PC discussed the concepts of the manuscript. MQ drew the figures. MQ, XY, XW, HZ and YS approved the version to be submitted. All authors contributed to the article and approved the submitted version.

## Glossary

**Table:**

cGAS	cyclic GMP-AMP synthase
COVID-19	coronavirus disease 2019
DARPins	designed ankyrin repeat proteins
dsRNA	double-stranded RNA
GSK-3β	glycogen synthase kinase-3β
HATs	histone acetyltransferases
HBV	hepatitis B virus
HCC	hepatocellular carcinoma
HCV	hepatitis C virus
HDAC	histone deacetylase
HDAC6	histone deacetylase 6
HIV	human immunodeficiency virus
IAV	influenza A virus
IFN-I	type-I interferon
IFN-β	interferon-β
IL	interleukin
IRF3	interferon regulatory factor 3
ISG	interferon-stimulated gene
MAVS	mitochondrial anti-viral-signaling protein
MTs	Microtubules
MyD88	myeloid differentiation primary response 88
NF-κB	nuclear factor kappa B
NLRP3	NACHT, LRR, and PYD domains-containing protein
oHSV	oncolytic herpes simplex virus
PA	polymerase acidic
PAMPs	pathogen-associated molecular patterns
PCV	porcine circoviruses
PKC	Protein kinase C
PRRs	pattern recognition receptors
PTM	post-translational modification
RIG-I	retinoic acid-inducible gene-I
RLRs	RIG-I-like receptors
SeV	sendai virus
SGs	stress granules
TBK1	TANK-binding kinase 1
TDP-43	transactive response DNA-binding protein
TLRs	toll-like receptors
TRIM21	tripartite motif-containing protein 21
VSV	vesicular stomatitis virus
ZnF-UBP	zinc-finger ubiquitin-specific protease
